# Trends in Breast Cancer Incidence, by Race, Ethnicity, and Age Among Women Aged ≥20 Years — United States, 1999–2018

**DOI:** 10.15585/mmwr.mm7102a2

**Published:** 2022-01-14

**Authors:** Taylor D. Ellington, Jacqueline W. Miller, S. Jane Henley, Reda J. Wilson, Manxia Wu, Lisa C. Richardson

**Affiliations:** ^1^Division of Cancer Prevention and Control, National Center for Chronic Disease Prevention and Health Promotion, CDC; ^2^Oak Ridge Institute for Science and Education, Oak Ridge, Tennessee.

Breast cancer is commonly diagnosed among women, accounting for approximately 30% of all cancer cases reported among women.* A slight annual increase in breast cancer incidence occurred in the United States during 2013–2017 (*1*). To examine trends in breast cancer incidence among women aged ≥20 years by race/ethnicity and age, CDC analyzed data from U.S. Cancer Statistics (USCS) during 1999–2018. Overall, breast cancer incidence rates among women decreased an average of 0.3% per year, decreasing 2.1% per year during 1999–2004 and increasing 0.3% per year during 2004–2018. Incidence increased among non-Hispanic Asian or Pacific Islander women and women aged 20–39 years and decreased among non-Hispanic White women and women aged 50–64 and ≥75 years. The U.S. Preventive Services Task Force currently recommends biennial screening mammography for women aged 50–74 years (*2*). These findings suggest that women aged 20–49 years might benefit from discussing potential breast cancer risk and ways to reduce risk with their health care providers. Further examination of breast cancer trends by demographic characteristics might help tailor breast cancer prevention and control programs to address state- or county-level incidence rates^†^ and help prevent health disparities.

USCS includes incidence data from central cancer registries reporting to CDC’s National Program of Cancer Registries and National Cancer Institute’s Surveillance, Epidemiology, and End Results (SEER) Program.^§^ All malignant cases of breast cancer^¶^ diagnosed in women during 1999–2018 were selected from registries with high quality data covering 97% of the U.S. population.** Trends in breast cancer incidence per 100,000 U.S. 2000 standard population were examined for women aged ≥20 years by race/ethnicity for five mutually exclusive groups (non-Hispanic American Indian or Alaska Native, non-Hispanic Asian or Pacific Islander, non-Hispanic Black, Hispanic, and non-Hispanic White) and age group (20–39, 40–49, 50–64, 65–74, and ≥75 years). Annual percent change (APC) and average annual percent change (AAPC) in incidence were estimated using joinpoint regression, with a maximum of three joinpoints (up to four-line segments) allowed.^††^ Two-sided statistically significant differences from zero were determined using a *t*-test for APCs and AAPCs from linear regressions with zero joinpoints and a z-test for AAPCs from linear regressions with one or more joinpoints. APC and AAPC were considered to be >0 or <0 if p<0.05, otherwise, rates were considered stable. Incidence rates were calculated with SEER*Stat software (version 8.3.8; National Cancer Institute) and APC and AAPC were calculated in Joinpoint software (version 4.6.00; National Cancer Institute).^§§^ This activity was reviewed by CDC and was conducted consistent with applicable federal law and CDC policy.^¶¶^

During 1999–2018, breast cancer incidence among women aged ≥20 years decreased an average of 0.3% per year, decreasing 2.1% per year during 1999–2004 and increasing 0.3% per year during 2004–2018 (Table). Incidence trends varied by racial and ethnic group (Figure 1). Incidence among non-Hispanic White women, among whom rates were highest, decreased an average of 0.3% per year from 198.0 to 186.5 per 100,000 population, decreasing 2.3% per year during 1999–2004 and increasing 0.4% per year during 2004–2018. Incidence among non-Hispanic Black women did not change significantly during 1999–2018. Incidence among Hispanic women decreased an average of 1.6% per year during 1999–2004, then increased an average of 0.4% per year during 2004–2018. Incidence among non-Hispanic American Indian or Alaska Native women increased an average of 1.4% per year during 1999–2016 then stabilized during 2016–2018. Among non-Hispanic Asian or Pacific Islander women, incidence was stable during 1999–2005 and increased 1.4% per year during 2005–2018, increasing an average of 0.8% per year during 1999–2018.

**TABLE:** Number, rate, and change in rate* of breast cancer incidence^†^ among women aged ≥20 years, by race/ethnicity^§^ and age group — United States, 1999–2018

Characteristic	No.	1999 rate*	2018 rate*	Absolute change in rate	Year range	APC	AAPC 1999–2018
**Overall**	**4,290,123**	**189.3**	**177.8**	**−11.5**	**1999–2004**	**−2.1^¶^**	−0.3**
					**2004–2018**	**0.3^¶^**	
**Race/Ethnicity**							
AI/AN, non-Hispanic	20,325	121.4	127.3	5.9	1999–2016	1.4^¶^	0.6
					2016–2018	−6.6	
A/PI, non-Hispanic	145,751	122.4	143.5	21.1	1999–2005	−0.4	0.8**
					2005–2018	1.4^¶^	
Black, non-Hispanic	451,788	167.4	174.0	6.6	1999–2005	−0.1	0.3
					2005–2008	2.2	
					2008–2015	0.5	
					2015–2018	−1.3	
Hispanic	305,075	136.3	134.0	−2.3	1999–2004	−1.6^¶^	−0.1
					2004–2018	0.4^¶^	
White, non-Hispanic	3,341,855	198.0	186.5	−11.5	1999–2004	−2.3^¶^	−0.3**
					2004–2018	0.4^¶^	
**Age group, yrs**							
20–39	204,345	27.0	28.1	1.1	1999–2010	0.1	0.3**
					2010–2018	0.7^¶^	
40–49	659,045	154.1	160.5	6.4	1999–2002	−1.1	0.2
					2002–2018	0.4^¶^	
50–64	1,524,658	310.2	267.8	−42.4	1999–2005	−2.8^¶^	−0.9**
					2005–2018	0.0	
65–74	995,279	444.4	445.5	1.1	1999–2004	−2.8^¶^	0.0
					2004–2013	1.5^¶^	
					2013–2018	0.0	
≥75	906,796	460.5	406.9	−53.6	1999–2004	−2.4^¶^	−0.7**
					2004–2009	0.6	
					2009–2018	−0.4^¶^	

**Abbreviations:** AAPC = average annual percent change; AI/AN = American Indian or Alaska Native; APC = annual percent change; A/PI = Asian or Pacific Islander.

* Per 100,000 population; overall rates were age-adjusted to the 2000 U.S. standard population. AAPC and APC were calculated using joinpoint regression, which allowed different slopes for four periods; the year at which slopes changed could vary by age and race/ethnicity.

^†^ Cancer incidence data were compiled from cancer registries that meet U.S. Cancer Statistics data quality criteria (https://www.cdc.gov/cancer/npcr/standards.htm), covering 97% of the U.S. population.

**^§^** Mutually exclusive racial/ethnic groups are based on information about race/ethnicity that was collected separately and combined for this report. Race/ethnicity were grouped as non-Hispanic AI/AN, non-Hispanic A/PI, non-Hispanic Black, Hispanic, and non-Hispanic White. Hispanic persons can be any race. Data are not presented for those with unknown or other race or unknown ethnicity (n = 25,329).

^¶^ APC was significantly different from zero at the α = 0.05 level.

** AAPC was significantly different from zero at the α = 0.05 level.

**FIGURE 1:**
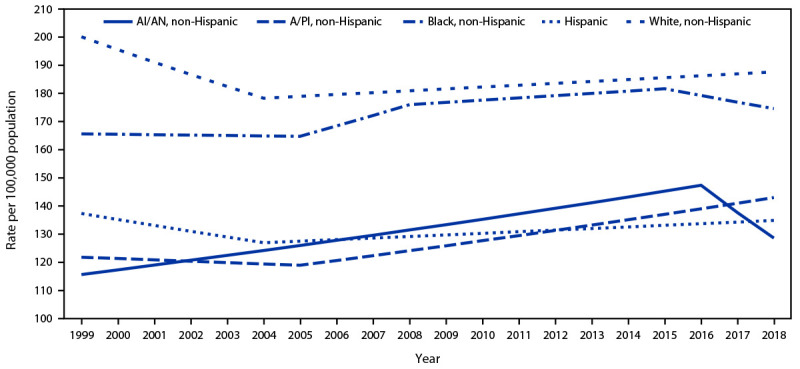
Trends* in breast cancer incidence^†^ among women aged ≥20 years, by race/ethnicity^§^^,^^¶^ ─ United States, 1999–2018 **Abbreviations:** AAPC = average annual percent change; AI/AN = American Indian or Alaska Native; A/PI = Asian or Pacific Islander. * Trends were estimated using joinpoint regression, with a maximum of three joinpoints (up to four-line segments) allowed; the year at which slopes changed could vary by age and race/ethnicity. Data displayed are the modeled age-adjusted rates. ^†^ Cancer incidence data were compiled from cancer registries that meet U.S. Cancer Statistics data quality criteria (https://www.cdc.gov/cancer/npcr/standards.htm), covering 97% of the U.S. population. ^§^ Mutually exclusive racial/ethnic groups are based on information about race/ethnicity that was collected separately and combined for this report. Race/ethnicity were grouped as non-Hispanic AI/AN, non-Hispanic A/PI, non-Hispanic Black, Hispanic, and non-Hispanic White. Hispanic persons can be any race. Data are not presented for those with unknown or other race or unknown ethnicity. ^¶^ AAPC was significantly different from zero at the α = 0.05 level for non-Hispanic A/PI and non-Hispanic White persons.

Among women aged <50 years, breast cancer incidence increased 0.7% per year during 2010–2018 among those aged 20–39 years and 0.4% per year during 2002–2018 among those aged 40–49 years (Table). In contrast, incidence among women aged 50–64 years stabilized during 2005–2018 after decreasing 2.8% per year during 1999–2005. Incidence among women aged 65–74 years decreased 2.8% per year during 1999–2004, increased 1.5% per year during 2004–2013, then stabilized during 2013–2018. Incidence among women aged ≥75 years decreased 2.4% per year during 1999–2004, was stable during 2004–2009, then decreased 0.4% per year during 2009–2018.

Among women aged 20–39 and 40–49 years, incidence was stable among non-Hispanic Black and Hispanic women but increased for other racial and ethnic groups (Figure 2). Incidence among non-Hispanic White women aged 50–64 years decreased an average of 0.8% per year, the largest decrease among any race/ethnicity and age group. Incidence among non-Hispanic American Indian or Alaska Native women aged 40–49 years increased an average of 1.9% per year, the largest increase among any racial/ethnic and age group.

**FIGURE 2:**
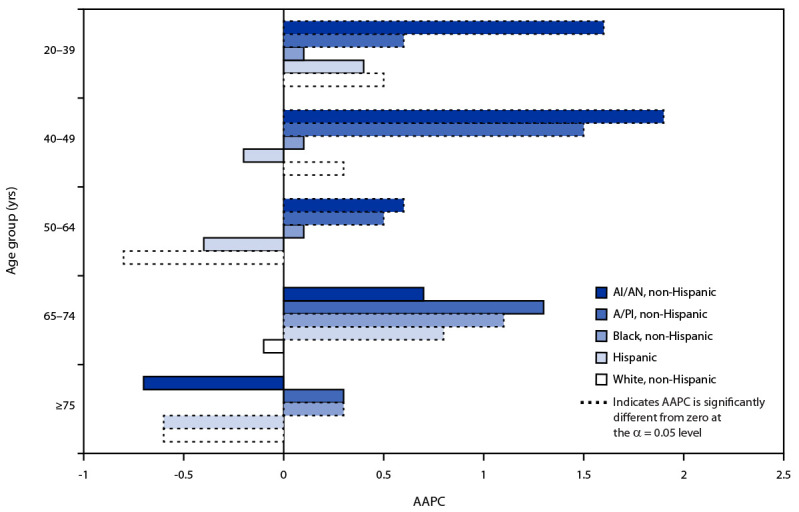
Average annual percent change* in breast cancer incidence^†^ among women aged ≥20 years by race/ethnicity^§^ and age group ─ United States, 1999–2018 **Abbreviations**: AAPC = average annual percent change; AI/AN = American Indian or Alaska Native; A/PI = Asian or Pacific Islander. * AAPC is the weighted average of the annual percent change during 1999–2018. To determine whether AAPC was significantly different from zero, a *t*-test was used if the joinpoint regression model had zero joinpoints, and a z-test was used if the joinpoint regression model had ≥1 joinpoint. ^†^ Cancer incidence data were compiled from cancer registries that meet U.S. Cancer Statistics data quality criteria (https://www.cdc.gov/cancer/npcr/standards.htm), covering 97% of the U.S. population. ^§^ Mutually exclusive racial/ethnic groups are based on information about race/ethnicity that was collected separately and combined for this report. Race/ethnicity were grouped as non-Hispanic AI/AN, non-Hispanic A/PI, non-Hispanic Black, Hispanic, and non-Hispanic White. Hispanic persons can be any race. Data are not presented for those with unknown or other race or unknown ethnicity.

## Discussion

The findings in this report indicate that breast cancer incidence among women aged ≥20 years decreased during 1999–2004 but increased during 2004–2018. During 1999-2018, incidence increased among non-Hispanic Asian or Pacific Islander women and women aged 20–39 years but decreased among non-Hispanic White women and women aged 50–64 and ≥75 years.

A previous study found that breast cancer incidence increased during 2004–2013 among Asian or Pacific Islander women, driven by a significant increase among those aged 45–49 years (*3*). Another study found that among women aged <45 years born in California, breast cancer risk among Asian or Pacific Islander women exceeded that among White women (*4*). Further examination of breast cancer incidence by detailed Asian or Pacific Islander race groups, age, cancer stage, and migration status might help further explain the increase in observed rates.

Results of this study also show that breast cancer incidence during 2010–2018 increased among women aged 20–49 years. Age and genetic, hormonal, and reproductive factors contribute to breast cancer risk. Modifiable risk factors for breast cancer include excess body weight (among postmenopausal women), physical inactivity, alcohol use, and hormone replacement therapy use.*** During 2017–2018, approximately 39.7% of women aged 20–39 years in the United States had obesity (body mass index ≥30 kg/m^2^), compared with 20.7% during 1988–1994, and a similar increase was observed during this period among women aged 40–59 and ≥60 years (*5*). The Community Preventive Services Task Force recommends evidence-based strategies to create social and physical environments that support healthy behaviors, such as reduced excessive alcohol use and increased physical activity (*6*). CDC’s National Comprehensive Cancer Control Program assists programs to help support and promote these strategies in communities.^†††^ CDC’s Bring Your Brave campaign provides information about breast cancer to women aged <45 years.^§§§^

Some forms of hormone replacement therapy taken for >5 years during menopause can raise breast cancer risk. Previous studies have associated the observed decrease in breast cancer incidence, specifically in the early 2000s, to be temporally related to the first report of the Women’s Health Initiative (*7*). The report found an increased risk of breast cancer associated with hormone replacement therapy followed by a decrease in the use of hormone replacement therapy among postmenopausal women in the United States (*7*). In 2017, the North American Menopause Society announced that “for women aged younger than 60 years or who are within 10 years of menopause onset and have no contraindications, the benefit-risk ratio is most favorable for treatment of bothersome vasomotor symptoms and for those at elevated risk for bone loss or fracture” (*8*)*.* Women who receive hormone therapy for >5 years might need to monitor any symptoms associated with breast cancer and consult with their health care provider if any symptoms of breast cancer are noticed.

From 2008 to 2015, breast cancer screening increased slightly among Hispanic women but declined among other groups, including >10% in some groups, including Asian women (*9*). The U.S. Preventive Services Task Force recommends that women aged 50–74 years who are at average risk for breast cancer get a mammogram every 2 years (*2*). Women aged 40–49 years, particularly those who have a known first-degree relative (i.e., parent, child, or sibling) with breast cancer, should talk to their physician or other health care professionals about starting screening with mammography (*2*). CDC’s National Breast and Cervical Cancer Early Detection Program provides breast and cervical cancer screenings and diagnostic services to low-income, uninsured, and underinsured women across the United States.^¶¶¶^

The findings of this report are subject to at least two limitations. First, analyses based on race/ethnicity might be biased if race/ethnicity were systematically misclassified. However, ongoing efforts are made to ensure that this information is as accurate as possible.**** Finally, delays in cancer reporting might result in an underestimation of incidence.

In this report, trends in breast cancer incidence differed by demographic characteristics, suggesting that breast cancer prevention and control programs be tailored to address state- or county-level incidence rates and help prevent health disparities. Breast cancer risk can be reduced with healthy behaviors, including maintaining a healthy weight, engaging in regular physical activity, and reducing alcohol use. The U.S. Preventive Services Task Force currently recommends biennial screening mammography for women aged 50–74 years (*2*); in addition, these findings suggest women aged 20–49 years might benefit from discussions with their health care providers about potential breast cancer risk and ways to reduce risk.

**Summary**

**What is already known about this topic?**

Breast cancer accounts for 30% of all cancers diagnosed in women.

**What is added by this report?**

During 1999–2018, breast cancer incidence among women aged ≥20 years decreased an average of 0.3% per year, decreasing 2.1% per year during 1999–2004 and increasing 0.3% per year during 2004–2018. Incidence increased among non-Hispanic Pacific Islander women and women aged 20–39 years but decreased among non-Hispanic White women and women aged 50–64 and ≥75 years.

**What are the implications for public health practice?**

The U.S. Preventive Services Task Force currently recommends biennial mammography screening for women aged 50–74 years. Women aged 20–49 years might benefit from discussing potential breast cancer risk and ways to reduce risk with their health care providers.

## Footnotes

*
https://www.cdc.gov/cancer/dataviz

†
https://gis.cdc.gov/Cancer/USCS/#/StateCounty/

§
https://www.cdc.gov/cancer/uscs/index.htm

¶
http://www.iacr.com.fr/index.php?option=com_content&view=category&layout=blog&id=100&Itemid=577

**
https://www.cdc.gov/cancer/uscs/technical_notes/criteria/registries.htm

†† Annual percent change and average annual percent change calculated in Joinpoint software (version 4.6.00; National Cancer Institute).

§§ Incidence and death rates calculated in SEER*Stat software (version 8.3.8; National Cancer Institute).

¶¶ 45 C.F.R. part 46.102(l)(2), 21 C.F.R. part 56; 42 U.S.C. Sect. 241(d); 5 U.S.C. Sect. 552a; 44 U.S.C. Sect. 3501 et seq.

***
https://www.cdc.gov/cancer/breast/basic_info/risk_factors.htm

†††
https://www.cdc.gov/cancer/ncccp/

§§§
https://www.cdc.gov/cancer/breast/young_women/bringyourbrave/take_action/index.htm

¶¶¶
https://www.cdc.gov/cancer/nbccedp/screenings.htm

****
https://www.cdc.gov/cancer/uscs/technical_notes/interpreting/race.htm
