# *Erratum:* Vol. 71, No. 2

**DOI:** 10.15585/mmwr.mm7104a5

**Published:** 2022-01-28

In the report “Trends in Breast Cancer Incidence, by Race, Ethnicity, and Age Among Women Aged ≥20 Years — United States, 1999–2018,” on page 47, in the Summary box, the second sentence in the “What is added by this report?” section should have read, “Incidence increased among non-Hispanic **Asian or** Pacific Islander women and women aged 20–39 years but decreased among non-Hispanic White women and women aged 50–64 and ≥75 years.”

